# Severe Fever with Thrombocytopenia Syndrome Phlebovirus causes lethal viral hemorrhagic fever in cats

**DOI:** 10.1038/s41598-019-48317-8

**Published:** 2019-08-19

**Authors:** Eun-sil Park, Masayuki Shimojima, Noriyo Nagata, Yasushi Ami, Tomoki Yoshikawa, Naoko Iwata-Yoshikawa, Shuetsu Fukushi, Shumpei Watanabe, Takeshi Kurosu, Michiyo Kataoka, Akiko Okutani, Masanobu Kimura, Koichi Imaoka, Kenichi Hanaki, Tadaki Suzuki, Hideki Hasegawa, Masayuki Saijo, Ken Maeda, Shigeru Morikawa

**Affiliations:** 1 Department of Veterinary Science, National Institute of Infectious Diseases, Tokyo, Japan; 2 Department of Virology 1, National Institute of Infectious Diseases, Tokyo, Japan; 3 Department of Pathology, National Institute of Infectious Diseases, Tokyo, Japan; 4 Division of Experimental Animal Research, National Institute of Infectious Diseases, Tokyo, Japan; 5 Laboratory of Veterinary Microbiology, Yamaguchi University, Yamaguchi, Japan

**Keywords:** Viral transmission, Viral host response, Viral pathogenesis

## Abstract

Severe fever with thrombocytopenia syndrome (SFTS) is an emerging hemorrhagic fever caused by the SFTS phlebovirus (SFTSV). SFTS patients were first reported in China, followed by Japan and South Korea. In 2017, cats were diagnosed with SFTS for the first time, suggesting that these animals are susceptible to SFTSV. To confirm whether or not cats were indeed susceptible to SFTSV, animal subjects were experimentally infected with SFTSV. Four of the six cats infected with the SPL010 strain of SFTSV died, all showing similar or more severe symptoms than human SFTS patients, such as a fever, leukocytopenia, thrombocytopenia, weight loss, anorexia, jaundice and depression. High levels of SFTSV RNA loads were detected in the serum, eye swab, saliva, rectal swab and urine, indicating a risk of direct human infection from SFTS-infected animals. Histopathologically, acute necrotizing lymphadenitis and hemophagocytosis were prominent in the lymph nodes and spleen. Severe hemorrhaging was observed throughout the gastrointestinal tract. B cell lineage cells with MUM-1 and CD20, but not Pax-5 in the lesions were predominantly infected with SFTSV. The present study demonstrated that cats were highly susceptible to SFTSV. The risk of direct infection from SFTS-infected cats to humans should therefore be considered.

## Introduction

Severe fever with thrombocytopenia syndrome (SFTS) is an emerging hemorrhagic fever caused by SFTS phlebovirus (SFTSV), which belongs to the genus *Phlebovirus* in the family *Phenuiviridae*, order *Bunyavirales*^1^. SFTS patients have been reported in China, Japan and South Korea, and the annual rates of reported cases have been increasing in these countries^2–4^.

SFTS is characterized by three stages according to the disease progress: a fever stage, a multiple organ dysfunction (MOD) stage and a convalescence stage. The fever stage is characterized by the sudden onset of a fever, headache and gastrointestinal (GI) symptoms^5^, showing thrombocytopenia, leukocytopenia, lymphadenopathy and high serum viral loads. The MOD stage is characterized by hemorrhagic manifestations, neurologic symptoms, sustained platelet decline, disseminated intravascular coagulation (DIC) and MOD. And patients with mild symptoms who survive SFTS then progress to the convalescence stage, where their condition begins to improve.

The risk factors for fatal cases have been determined to be an older age, high serum viral loads, substantial elevation of serum biochemical profiles (asparate aminotransferase (AST)/alanine aminotransferase (ALT), creatine kinase (CK), creatine kinase MB (CK-MB) and lactate dehydrogenase (LDH)), hemorrhagic symptoms, neurologic symptoms, DIC and MOD^5–8^.

Although the life cycle of SFTSV is still not fully understood, mammalian hosts and ticks are considered to play a crucial role in the maintenance of SFTSV in nature. In small- and large-scale molecular and serological surveys, low to high copies of viral RNA and antibodies against SFTSV have been detected in a variety of animals, both wild as well as domesticated, including dogs and cats^6,9–12^. However, many of these animals show no or obscure clinical signs, suggesting that animals can be subclinically infected with SFTSV.

Humans have traditionally been considered to be infected with SFTSV by tick bites; however, possible cases of person-to-person transmission of SFTSV have also been reported^13–16^. Furthermore, SFTS patients without histories of tick bite are also reported. Breeding domestic animals are reported to be a significant determinant of the SFTS seroprevalence in China^17^. In this respect, it is important to assess the risk of human infection of SFTSV from animals.

In 2017, cheetahs in a zoo^18^ and roaming and indoor cats were diagnosed with SFTS in epidemic areas of Japan. Until 2018, 80 cats were diagnosed with SFTSV infection and approximately 60% of them were died in Japan (personal communication from Dr. Ken Maeda). Since their symptoms, results of clinical analyses and pathological examination findings were similar to those of SFTS patients, the family *Felidae* seems to be susceptible to SFTS, similar to humans.

To confirm whether or not cats are indeed susceptible to SFTSV, we performed experimental infection of cats with SFTSV and assessed their outcomes.

## Results

### Clinical signs

**Figure 1:**
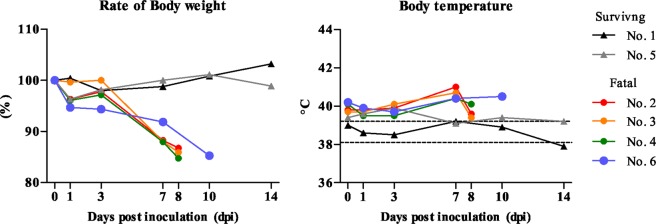
Clinical signs. Six cats were inoculated intravenously (i.v.) with SFTSV SPL010 strain (10^7^ TCID_50_/mL) (Table 3). Their body weight and body temperature were monitored under anesthetization for one month after inoculation.

Four of six cats infected with the SFTSV showed weight loss from 3 to 8 days post-inoculation (dpi), and the body temperature was the highest at 7 or 10 dpi (Fig. 1). They showed ruffled fur, anorexia, depression or aggressive behavior and salivation at 7 or 8 dpi and reached the humane endpoint. Their urine became dark orange from 6 dpi. The other two cats infected with SFTSV showed no obvious clinical signs during the experimental period.

**Figure 2:**
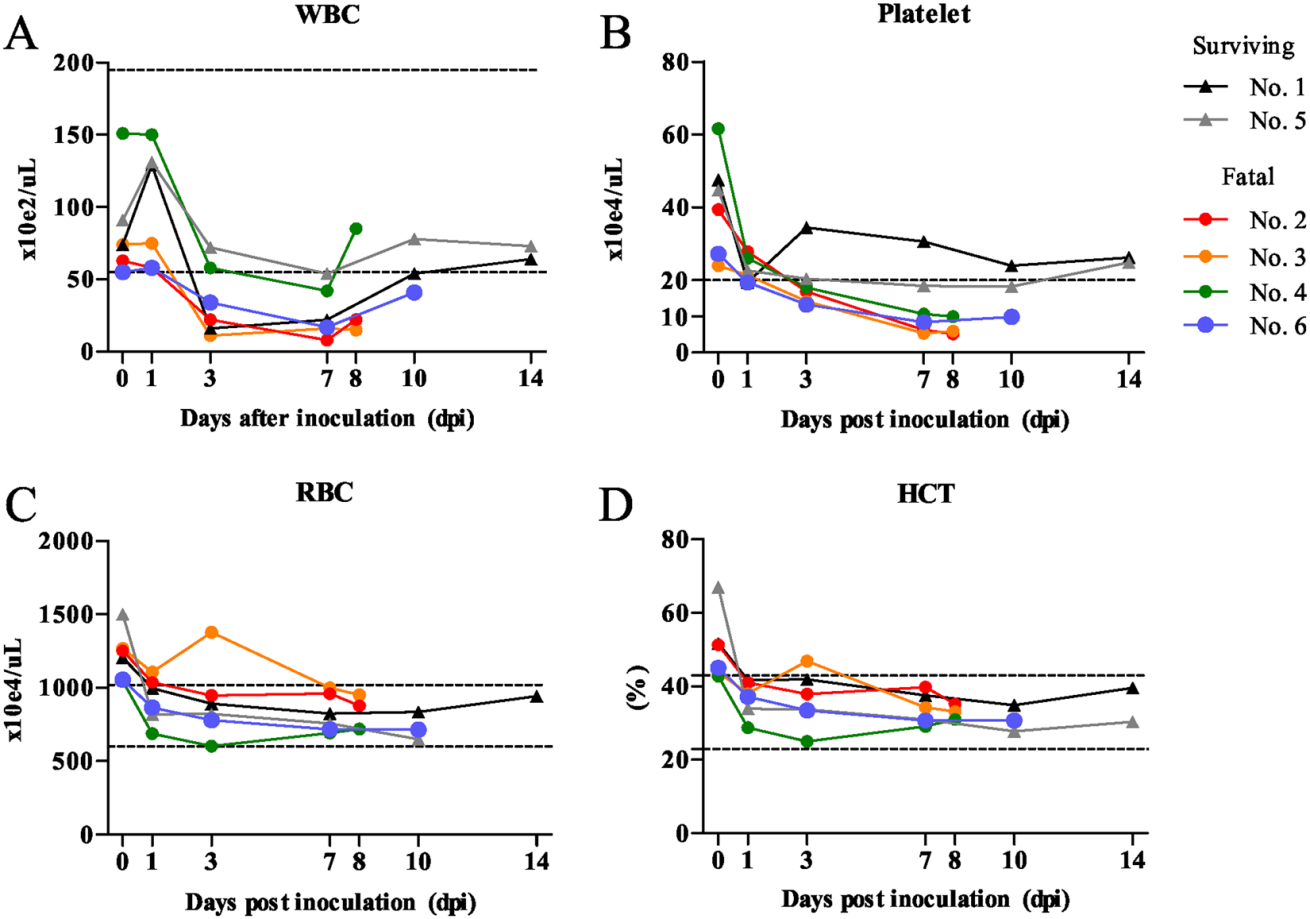
Blood cell count. Blood specimens were subjected to a complete blood cell count analysis using an automated blood cell counter. The WBC (**A**), Platelet (**B**), RBC (**C**) and hematocrit (HCT) were measured.

The four fatal cats infected with SFTSV developed leukopenia and thrombocytopenia according to an automated blood cell counter (Fig. 2). The white blood cell (WBC) count decreased from 3 to 7 dpi in the cats and two surviving cats (No. 1 and 5) recovered from 10 dpi (Fig. 2A). The platelet count decreased from 1 to 8 or 10 dpi in all cats; however, the decrease was particularly severe in the fatal cats (No. 2, 3, 4 and 6). In the surviving cats, the platelet count recovered from 14 dpi (Fig. 2B). The red blood cell (RBC) count and the hematocrit (HCT) level decreased slightly from 1 dpi in all cats (Fig. 2C,D), while the hemoglobin, mean corpuscular volume (MCV), mean corpuscular hemoglobin (MCH) and mean corpuscular hemoglobin concentration (MCHC) did not markedly decrease during the experimental period (data not shown), indicating that the cats did not show hemolytic anemia.

**Figure 3:**
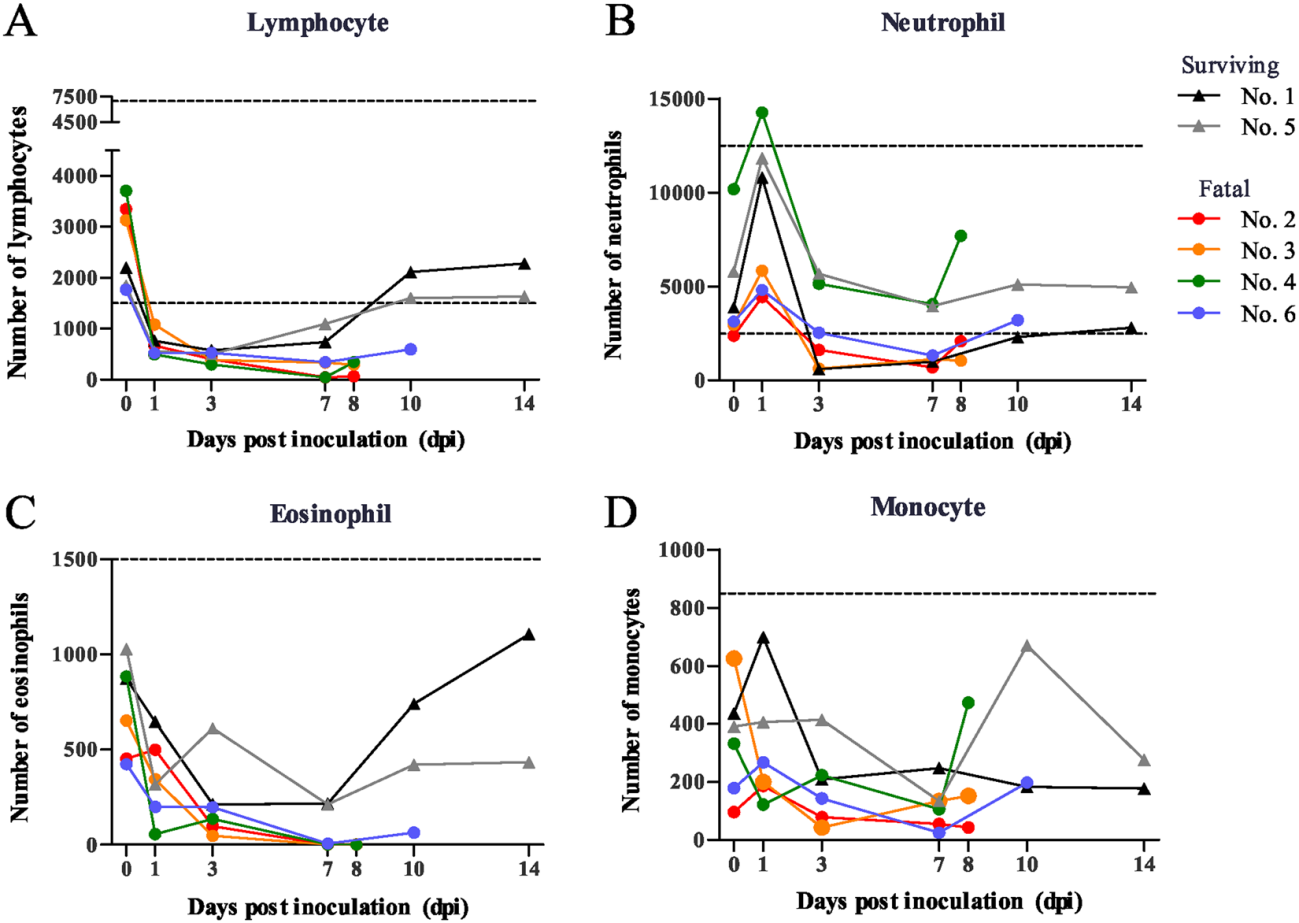
Differential counts of blood cells. Stained blood smears were examined to evaluate the lymphocytes (**A**), neutrophils (**B**), eosinophils (**C**) and monocytes (**D**). The ratio of cells at each dpi was calculated at based on the number of cells at day 0.

Regarding the leukocyte differential count, a blood cell count of lymphocytes, neutrophils, eosinophils and monocytes was performed using blood smear samples (Fig. 3). In the analysis, the ratio to the number of cells at day 0 was calculated. The ratio of lymphocytes, especially in the four fatal cats, markedly decreased from 1 dpi and was lowest at 7 dpi, 1% in the most fatal cats (No. 2 and 4) (Fig. 3A). The ratio of neutrophils, eosinophils and monocytes also decreased from 1 dpi in the cats (Fig. 3B–D). From these results, it was appeared that clinical symptoms showed from 3 dpi and the occurrence of leukopenia and thrombocytopenia reached the peak at 7 dpi in the fatal cats inoculated with SFTSV.

**Figure 4:**
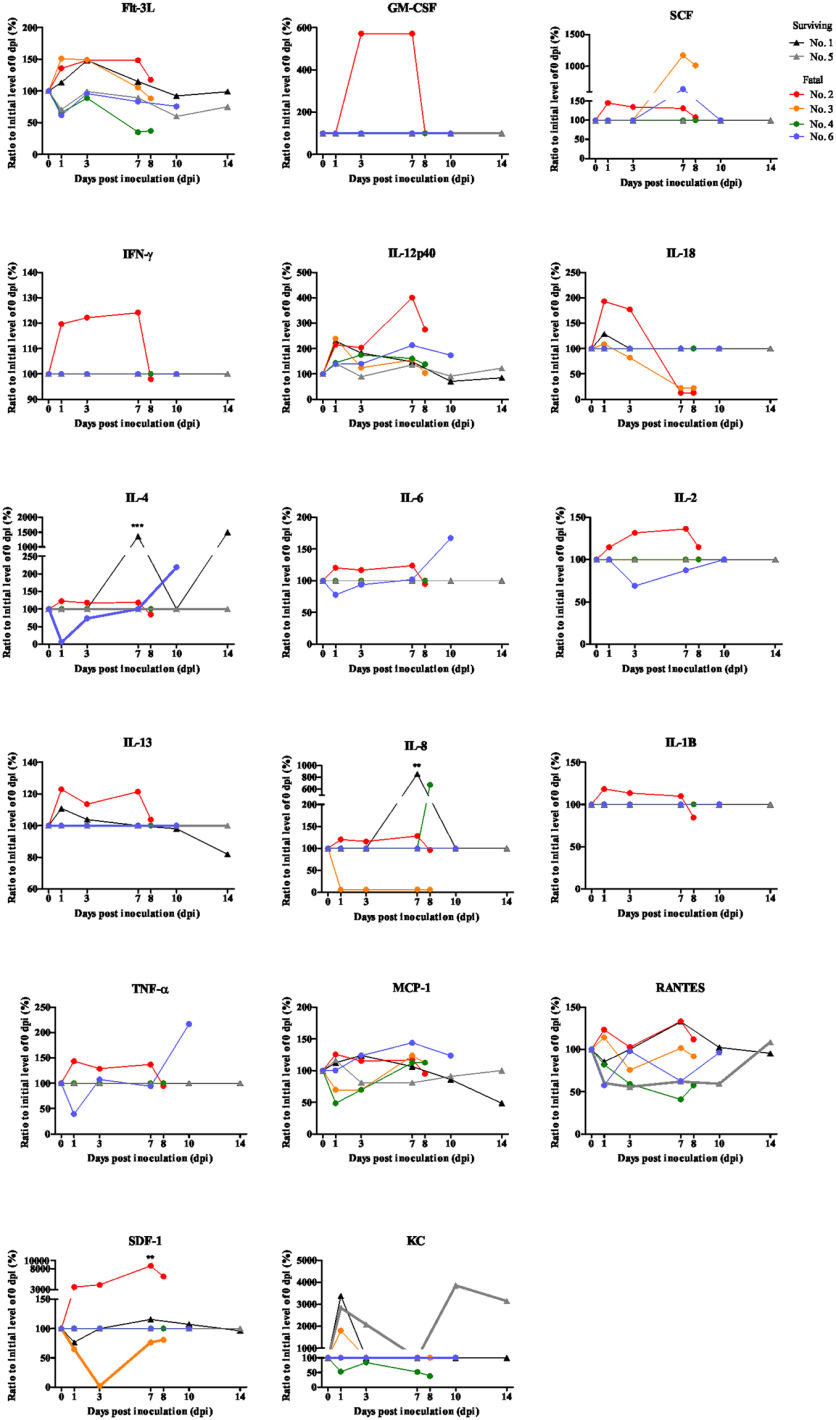
Cytokines and chemokines were measured by a multiplex assay.

The levels of cytokine and chemokine were determined by a multiplex assay (Fig. 4). The levels of cytokine and chemokine were determined by a multiplex assay (Fig. 4). The level of pro-inflammatory cytokines and chemokines, such as Flt-3L, GM-CSF, IFN-γ, IL-12p40, IL-4 (P < 0.005), IL-6, IL-8 (P < 0.005), RANTES and SDF-1 (P < 0.005), was elevated from 1 dpi and reached the peat at 7 dpi, then followed by the rapid reduction at 8 dpi in the most fatal cat (No. 2). The level of TNF-α and MCP-1 was the peak at 1 dpi. The regulatory T cell cytokine, IL-2, was elevated to 7 dpi and reduced at 8 dpi. The anti-inflammatory cytokine, IL-13, was the peak at 1 dpi and reduced at 8 dpi. These results indicated the stimulated or active immune status of the cat (No. 2). One fatal cat (No. 6) had an elevated level of IL-4 and IL-6 relating to B cell proliferation and immunoglobulin production at 10 dpi. The level of pro-inflammatory cytokines and chemokines, such as SCF, IL-12p40, TNF-α and MCP-1, were the peak at 7 or 10 dpi. Other fatal cats (No. 3 and 4) had no remarkable changes of cytokine or chemokine levels. These results indicated the cytokine and chemokine responses were varied among the SFTSV infected fatal cats.

**Figure 5:**
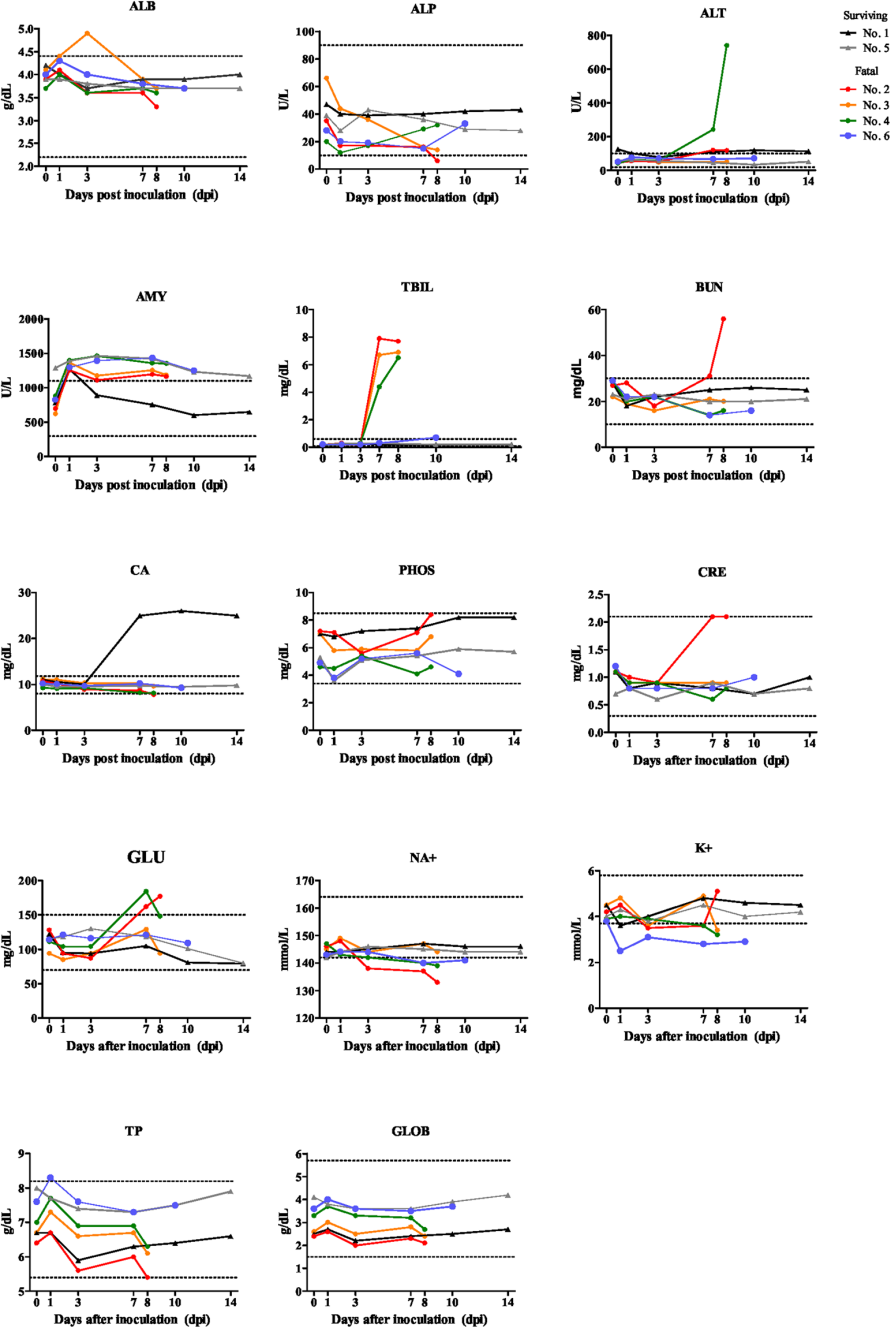
Biochemical analyses. The biochemical values of sera were measured using an automated analyzer. Albumin (ALB), alkaline phosphatase (ALP), alanine aminotransferase (ALT), (AMY), total bilirubin (TBIL), blood urea nitrogen (BUN), total calcium (CA), phosphorus (PHOS), creatinine (CRE), glucose (GLU), NA^+^, K^+^, total protein (TP) and globulin (GLOB) were determined. The dotted line in each graph shows the physiological reference of each matter.

The results of a biochemical examination of the cat sera are shown in Fig. 5. The values of ALP prominently decreased in the four fatal cats, especially in the most fatal cat (No. 2). The values of TBIL markedly elevated from 3 dpi and were the highest from 7 to 10 dpi, especially in the severe cats (No. 2, 3 and 4). The values of BUN and CRE were high in the most fatal cat (No. 2). Serum NA+ and K+ decreased at 7 or 8 dpi in fatal cats (No. 2, 4 and 6), supporting the insufficiency of renal function. There was an increase in the GLU of two fatal cats (No. 2 and 4). Indeed, in the urine strip test, the SFTSV-inoculated fatal cats showed significantly higher values for urobilinogen, bilirubin, protein and specific gravity (≥1.030) than the surviving cats (Table 1). Thus, these results indicated that the fatal cats had jaundice and hepatic or renal damage.

**Table 1:** Results of the urine examination.

	No. 1	No. 2	No. 3	No. 4	No. 5	No. 6
	Survived	Moribund	Moribund	Moribund	Survived	Moribund
Urobilinogen	0	1	1.5	0	0	1
Occult blood	0	1	0	0	2	0
Bilirubin	0	3	3	3	0	2
Keton body	0	0	0	0	0	0
Glucose	0	0	0	0	0	0
Protein	1	2	2	2	2	2
pH	6	6	6	6	6	6
Specific gravity	1.025	≧1.030	≧1.030	≧1.030	1.02	≧1.030

Taken together, these findings demonstrate that the fatal cats infected with SFTSV showed severe clinical manifestations, such as a high fever, gastrointestinal symptoms, leukopenia, thrombocytopenia and hepatic and renal damage, similar to human SFTS patients. Our results thus indicate that cats are indeed susceptible to SFTSV infection.

### Detection of SFTSV RNA by quantitative polymerase chain reaction (qPCR) and isolation of SFTSV

**Figure 6:**
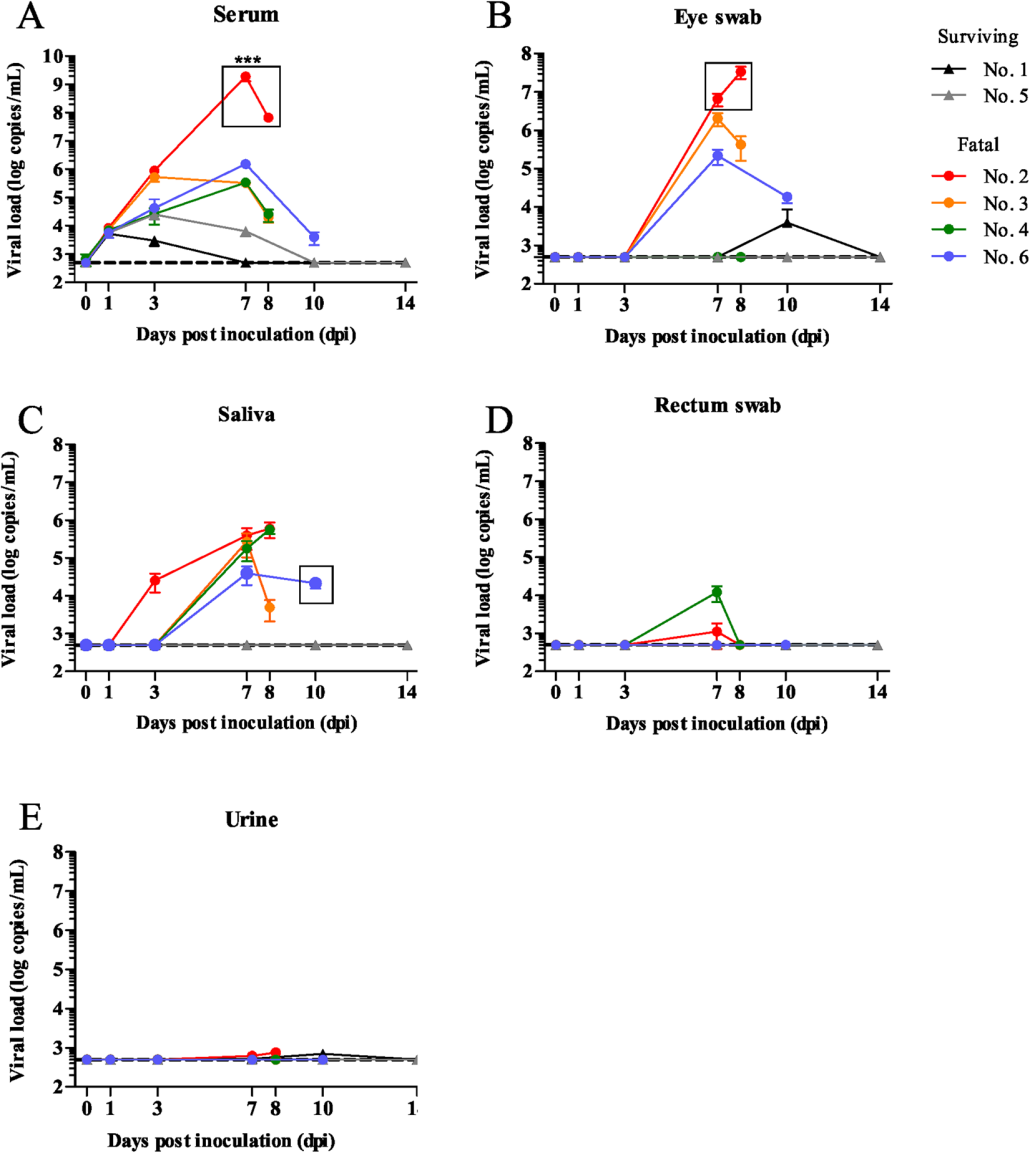
SFTS viral RNA copies. The number of viral RNA copies in serum, saliva, eye swab, rectal swab and urine samples was measured by qPCR. SFTSV was isolated from samples with high viral load (rectangles) ***P < 0.0001.

**Figure 7:**
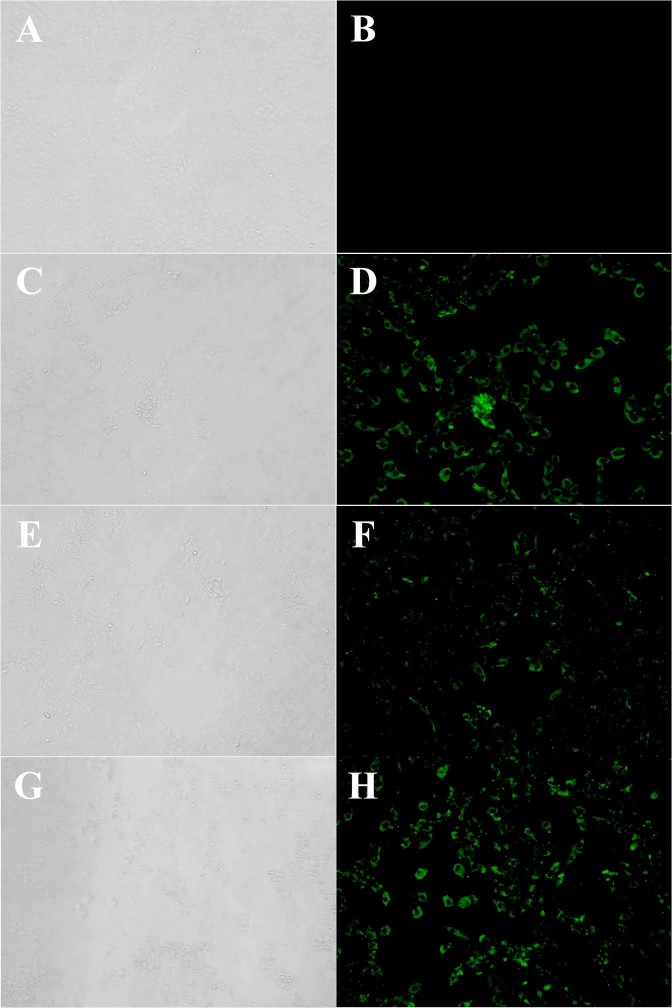
SFTSV isolation from samples. SFTSV was isolated from serum, eye swab and saliva and confirmed by IF. (**A,B**) Mock (Vero cells). (**C**,**D**) Eye swab (No. 2, 7 dpi). (**E**,**F**) Saliva (No. 6, 10 dpi). (**G**,**H**) Serum (No. 2, 7 dpi). Magnification of X20. (**A**,**C**,**E**,**G**), phase contrast image; (**B,D,F,H)**; IF staining.

The RNA copy numbers of SFTSV in the serum, saliva, urine and eye and rectal swab samples of the cats were measured by qRT-PCR (Fig. 6). The RNA copy numbers of SFTSV in the serum samples of the SFTSV-inoculated fatal cats were markedly higher (up to nearly 10^9^ RNA copies/mL) than those of other cats and significant at 7 dpi (P < 0.0001). High levels of SFTSV RNA were also detected in the eye swab and saliva samples of the SFTSV-inoculated fatal cats. Relatively low levels of SFTSV RNA were detected in the rectal swabs and urine of the cats. To confirm the infectious virus in the specimens, virus isolation was attempted from serum, saliva, urine, eye and rectal swab specimens collected during the experimental period. SFTSV was isolated from the serum, saliva and eye swab specimens with high SFTSV RNA copies (Fig. 6, rectangle and Fig. 7). The results showed high levels of viremia in the cats with fatal illness upon SFTSV infection, with the virus secreted via the blood, saliva and tears.

### IgM and IgG antibody responses and neutralizing antibody response in SFTSV-inoculated cats

**Figure 8:**
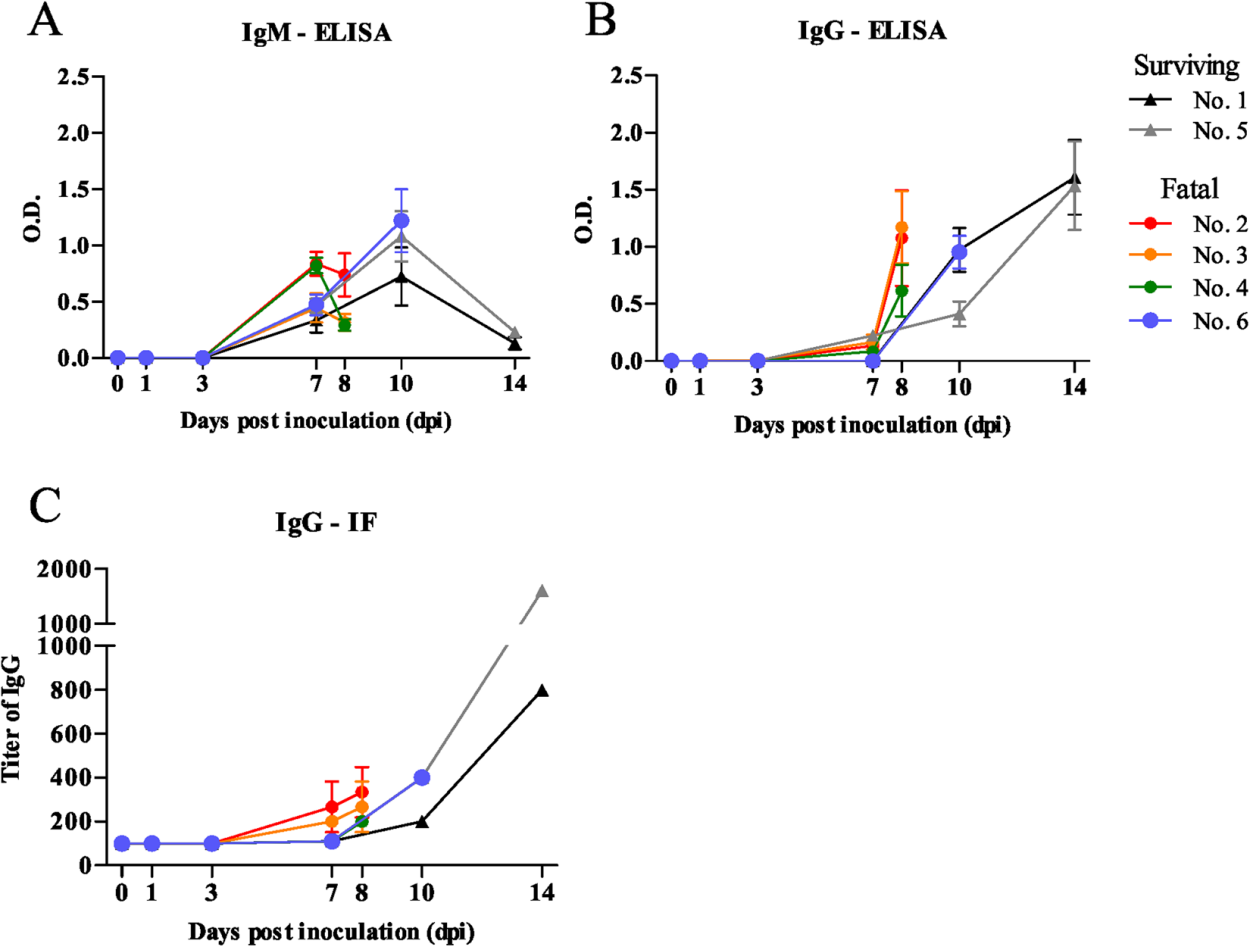
IgM and IgG antibody responses in the cats infected with SFTSV. (**A,B**) IgM and IgG antibodies against SFTSV were measured by an ELISA, and the OD of the serum dilution at 1:400 were shown. (**C**) Titer of IgG against SFTSV-NP were determined by IF. The limit of detection is 1:40.

IgM and IgG antibodies against SFTSV were measured by enzyme-linked immunosorbent assay (ELISA) and immunofluorescence (IF). The cut-off values of IgM and IgG-ELISA at a dilution of 1:400 were calculated as average OD values plus 3 × SD of the cats at 0 dpi and determined to be 0.17 and 0.16, respectively. IgM and IgG antibody responses at a serum dilution of 1:400 are shown (Fig. 8). IgM antibodies were detected at 7 dpi, reached at its peak at 10 dpi in the cats infected with SFTSV. IgG antibodies were first detected in the cats at 7 dpi, after which its levels increased. In addition, IgG antibodies against SFTSV were also confirmed by IF. In all cats, the level of IgG antibodies increased from 7 dpi in IF, similar to those in ELISA. Of note, the IgM and IgG antibody responses by ELISA and IF were not significantly different between the surviving and fatal cases (Fig. 8).

Then, serum samples were subjected to the neutralization test by determination of 50% plaque reduction neutralization titer (PRNT50). The neutralization titers of fatal cats were under the limit of detection (<10) or low (1:10) (Table 2). However, similar levels of neutralization titers were detected at 7 and 8 dpi but increased levels of neutralization titers were detected at 10 and 14 dpi in surviving cats. These indicated the cats were died before protective levels of neutralizing antibodies were induced in the fatal cases.

**Table 2:** Titer of neutralization antibodies of cats.

dpi	0 dpi	1 dpi	3 dpi	7 dpi	8 dpi	10 dpi	14 dpi	State of cat
No. of cat								
1	<10	<10	<10	10		20	>40	Surviving
2	<10	<10	<10	10	10			Fatal
3	<10	<10	<10	<10	<10			Fatal
4	<10	<10	<10	<10	10			Fatal
5	<10	<10	<10	10		>40	>40	Surviving
6	<10	<10	<10	<10		40		Fatal

### Pathological examinations

The four fatal SFTSV-inoculated cats became moribund and reached the humane endpoint between 8 and 10 dpi; they were considered to have a fatal course and were thus euthanized and autopsied. The pathological changes were evaluated grossly and microscopically. Simultaneously, the two surviving cats were euthanized and autopsied as controls at 14 dpi.

**Figure 9:**
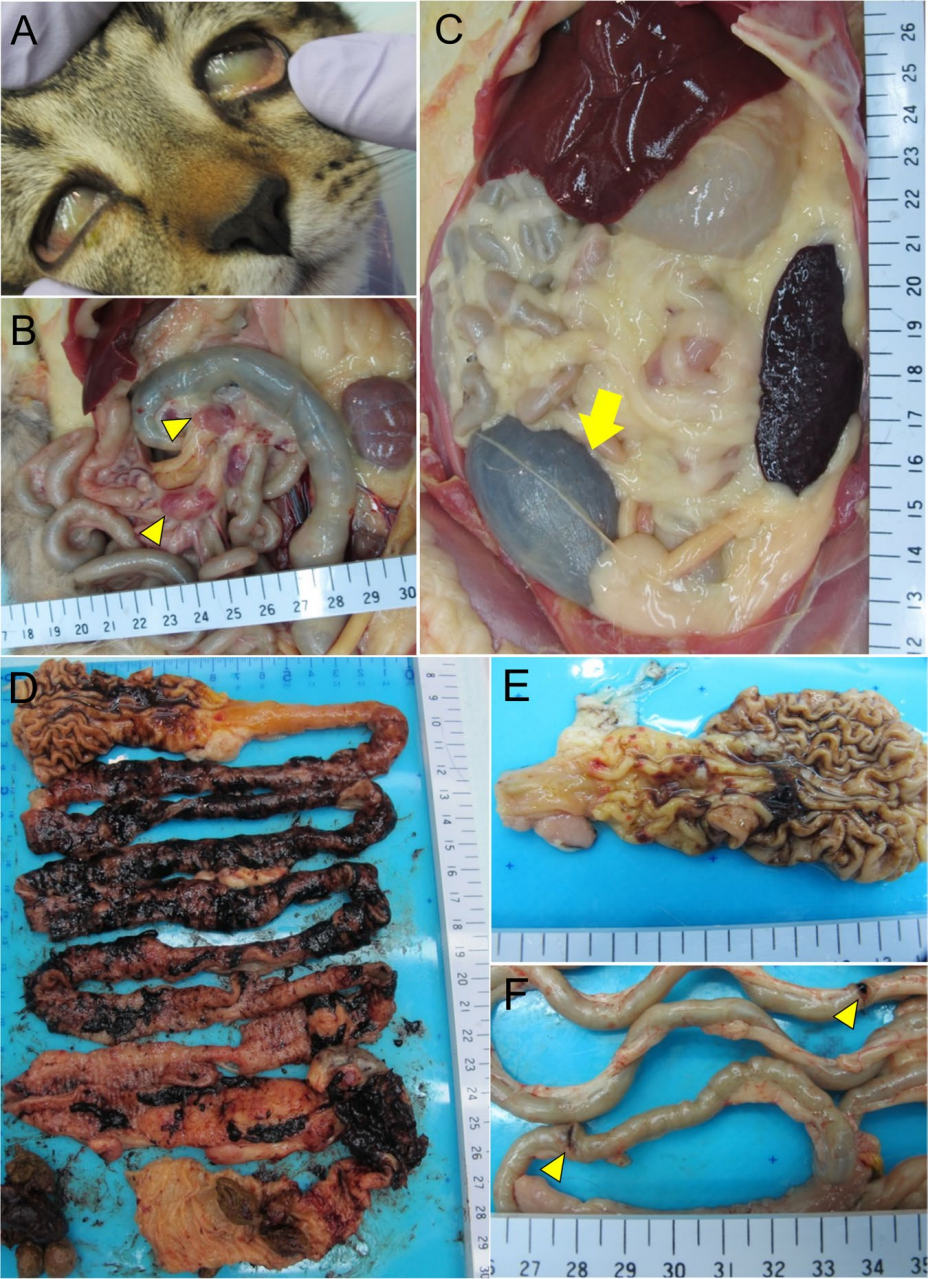
Gross lesions of cat No. 2 at 8 dpi with SFTSV. (**A**) Marked jaundice was shown in the sclera. (**B**) The intestinal LNs were reddish and swollen (yellow arrowhead). (**C**) The urinary bladder was filled with brown to black contents (yellow arrow). (**D**) Extensive hemorrhage was observed throughout the GI tract, which was filled with black mucous contents from the duodenum to rectum. (**E**) Considerable reddish to black hemorrhagic lesions were observed on the mucosal surface of stomach. (**F**) Perforation of the duodenal wall was observed (yellow arrowhead).

The four fatal cats showed yellowish discoloration of the conjunctiva, mucous membrane of the oral cavity, skin and fat tissue, indicating jaundice (Fig. 9A). The lymph nodes (LN)s, such as the cervical, submandibular, intestinal and inguinal LNs, were swollen (Fig. 9B). Some swollen LNs were reddish. The dark-red spleen was thin and had a hard surface (Fig. 9C). The urinary bladder was filled with brown to black urine (Fig. 9C). The most prominent gross lesions were observed in the gastrointestinal tract (Fig. 9D). There were marked reddish to black spots of gastric hemorrhage on the mucosal surface of the pyloric gland region (Fig. 9E). Hemorrhage was severe throughout the gastrointestinal tract, from the duodenum to the rectum, with black-colored mucous contents (Fig. 9D). In the most severe case, two sites of the duodenal wall were perforated (Fig. 9F). Peyer’s patches were recognized as red spots in the small intestine from the fatal animals.

**Figure 10:**
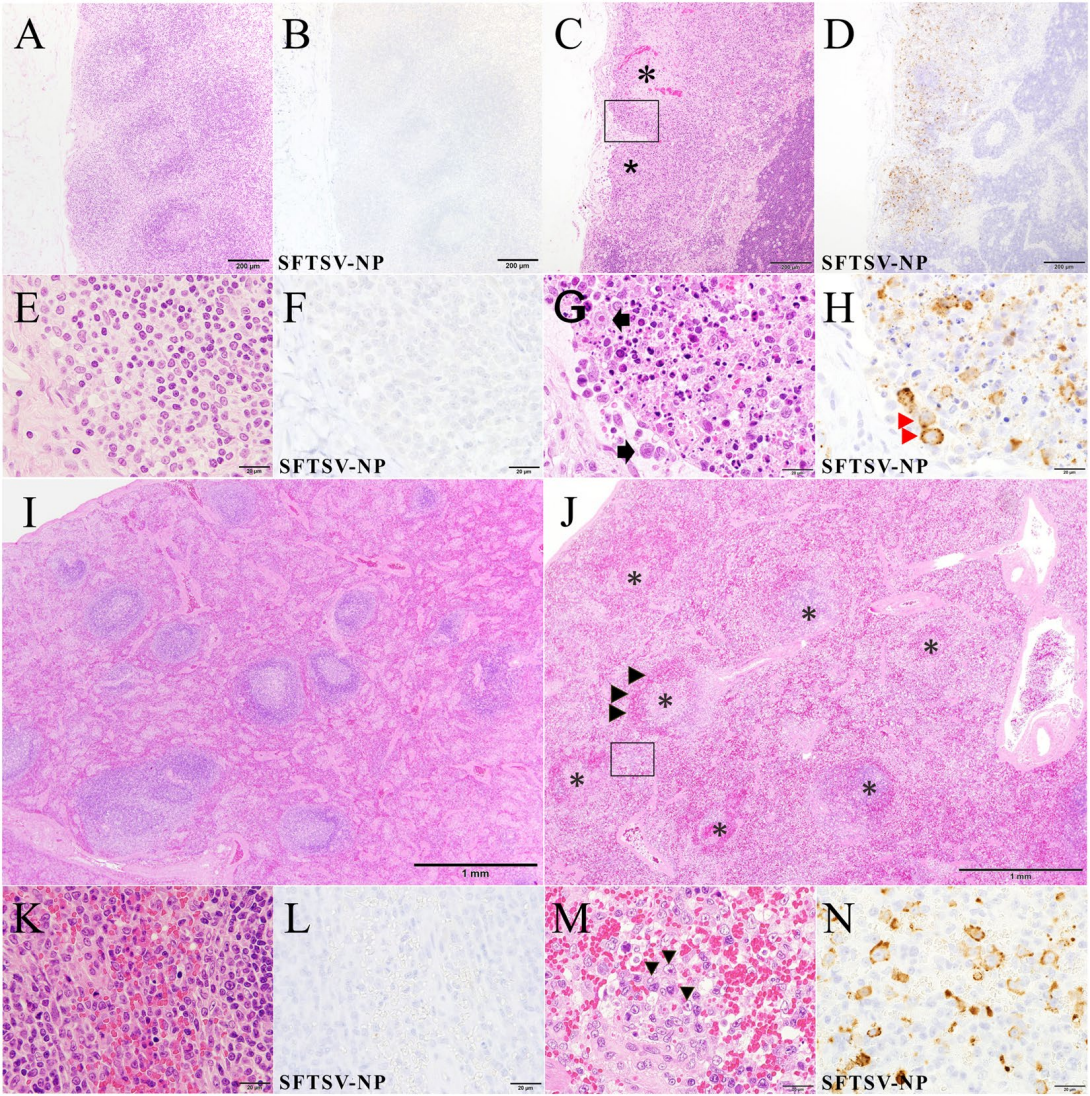
Cervical LNs and spleen of cat No. 1 and acute necrotizing lymphadenitis of the cervical LNs and spleen of cat No. 2 with SFTSV. (**A–H**), Cervical LN; (**I–N**), Spleen. (**A**) Normal structure of lymphatic nodules of surviving cat No. 1 are observed. (**B**) This area is negative for virus antigen. (**C**) The construct of the dark cortex is unclear because some lymphatic nodules are absent. The subcapsular sinus is enlarged, and lymphocytes are depleted in the nodule (*). Fatal cat No. 2. (**D**) Some areas in the cortex without lymphatic nodules are positive for virus antigen. Many SFTSV-NP-positive cells are seen in the lesion. (**E**) Normal cells of lymphatic nodules are observed. Cat No. 1. (**F**) These cells are negative for viral antigen. (**G**) Necrotizing lymphatic nodule (higher magnification of a boxed section of **C**). Necrotic cells with pyknosis and round-shaped mononuclear cells (arrows) are seen in the sinus and nodule. (**H**) The round-shaped cells and cell debris are positive for SFTSV-NP (red arrowheads). **(I**) Normal structure of white pulp and red pulp are observed. Cat No. 1. (**J**) Necrotic nodules of the white pulp of spleen are observed (*). Cat No. 2. The number of erythrocytes decreased in the red pulp while red cells are accumulated in the marginal parts of the nodule (arrowheads). (**K**) Normal structure of red pulp of the spleen is observed. Cat No. 1. (**L**) There are not SFTSV-NP-positive cells. (**M**) The red pulp of the spleen (higher magnification of a boxed section of **J**). Several round-shaped mononuclear cells are present. (**N**) The round-shaped mononuclear cells are SFTSV-NP-positive. (**A,C,E,G,I–K**,**M**): H&E staining, (**B,D,F,H,L**,**N)**: SFTSV-NP means SFTSV NP antigen stained by IHC. Bars, (**A–D)**, 200 µm; (**E–H**,**K–N)**, 20 µm; (**I,J)**, 1 mm.

**Figure 11:**
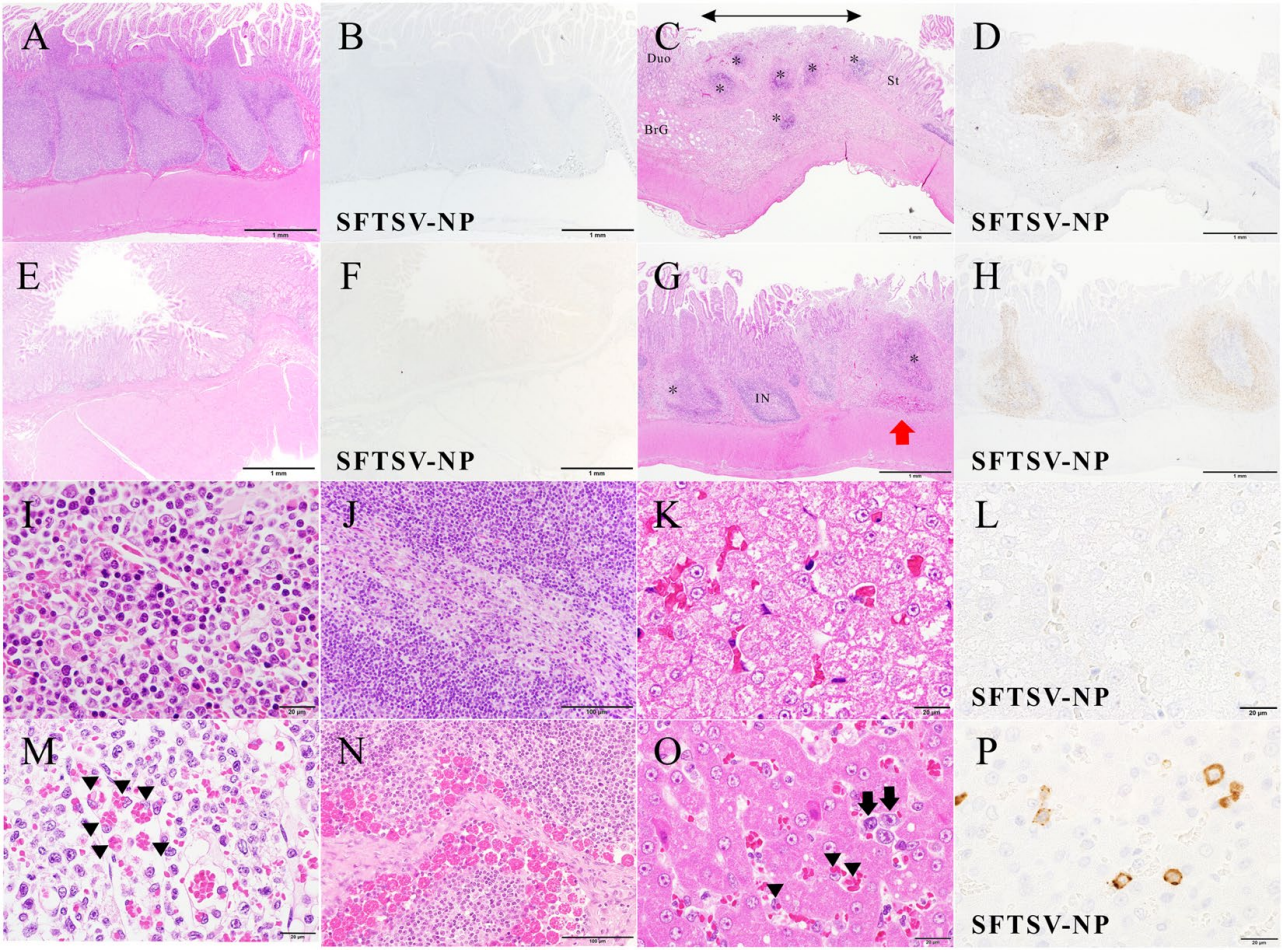
Hemophagocytosis and affected lymphatic nodules in the GI system of a fatal cat at 8 dpi with SFTSV and normal structures of surviving cat No. 1. (**A,B**) Normal aggregated lymphatic nodules in the lamina propria of the stomach of cat No. 1. SFTSV-NP-positive cells are not found. (**C**) Aggregated lymphatic nodules (*) in the lamina propria of the junction of pyloric gland region and duodenum. Decreased lymphocytes, necrotic germinal centers, congestion and missing nodule-associated epithelium (arrow) are seen in the lesions grossly identified as red spots in the stomach (in Fig. 8E). The villi are intact. St, stomach; Duo, duodenum; BrG, Brünner’s gland. (**D**) The lesional area was positive for SFTSV-NP. (**E,F**) Normal structure of the jejunum of cat No. 1. There are not found SFTSV-NP-positive cells. (**G,H**) Peyer’s patches in the jejunum. Necrotizing large sac-like lymphatic nodules (*) are positive for SFTSV-NP, while the internodular regions (IN) and villi are intact. Hemorrhaging occurred within the capsule of a nodule (red arrow). (**I–L**) Normal structures of surviving cat No. 1. I, sternal bone marrow; (**J**), mesenteric lymph node; (**K,L**), liver. (**M**) Hemophagocytosis in the sternal bone marrow from cat No. 2. Many hemophagocytic macrophages are present in the vascular space (arrowheads). (**N**) A mesenteric lymph node from cat No. 3. Numerous hemophagocytes are present in the medullary sinus. (**O**) Liver from cat No. 2. A large number of hemophagocytic macrophages (Kupffer cells, arrowheads) and round-shaped mononuclear cells (arrows) are seen in the sinusoids. (**P**) They are positive for SFTSV-NP (SFTSV-NP). The hepatocytes seem to be normal. (**A,C,E,G,I–O)**; H&E staining, (**C,D,F,H,P)**; SFTSV-NP, viral antigen by immunohistochemistry. Bars, (**A–H)**, 1 mm, (**I,K–M,O**,**P)**, 20 µm; (**J**,**N)**, 100 µm.

The histopathological changes were examined by hematoxylin and eosin (H&E) staining and double immunohistochemical staining (IHC) for viral antigens in the tissue samples. The main lesions were observed in the lymphatic tissues, including the LNs and spleen (Fig. 10) of fatal cats. The normal architecture of these tissues was replaced by marked necrosis with depletion of lymphocytes (Fig. 10). The infiltration of histiocytes, but not neutrophils, was predominant in the lesions, indicating acute necrotizing lymphadenitis. There were no histopathological findings of surviving cats (Fig. 10). CD20-positive cells decreased and granzyme B-positive cells infiltrated in the lesions of fatal cats extensively more than surviving cats. Necrotic cells showed significant apoptotic changes, including pyknosis, karyorrhexis and karyolysis (Fig. 10G). In these lesions, round-shaped mononuclear cells accumulated characteristically and were positive for SFTSV-NP antigen (Fig. 10D,H,N). The cells were morphologically similar to immunoblasts and larger than mature lymphocytes with bright nuclei and prominent eosinophilic nucleoli. Necrotic lymphatic nodules with SFTSV-NP antigen were also found in the mucosa-associated lymphatic tissues, such as the gut and respiratory tract (Fig. 11). The aggregated lymphatic nodules in the stomach, small intestine and cecum showed severe necrosis with hemorrhage, while the internodular regions and villi were intact. In addition, the fatal cats showed prominent hemophagocytosis in various tissues, including the spleen, bone marrow, LNs and liver (Fig. 11E–G). Contrary to fatal cats, surviving cats had no changes and SFTSV-NP antigens in these tissues (Fig. 11).

IHC showed that round-shaped immunoblast-like cells and cell debris in the lesions were predominantly SFTSV-NP antigen-positive (Fig. 10H,N). Some macrophages, not hemophagocytes, were also SFTSV-NP antigen-positive (Fig. 11P). Other organs, such as the heart, brain and kidney, had no obvious lesions in the parenchyma. However, SFTSV-NP-positive cells in the blood vessels of these organs were obvious in one fatal cat (No. 8) (data not shown).

**Figure 12:**
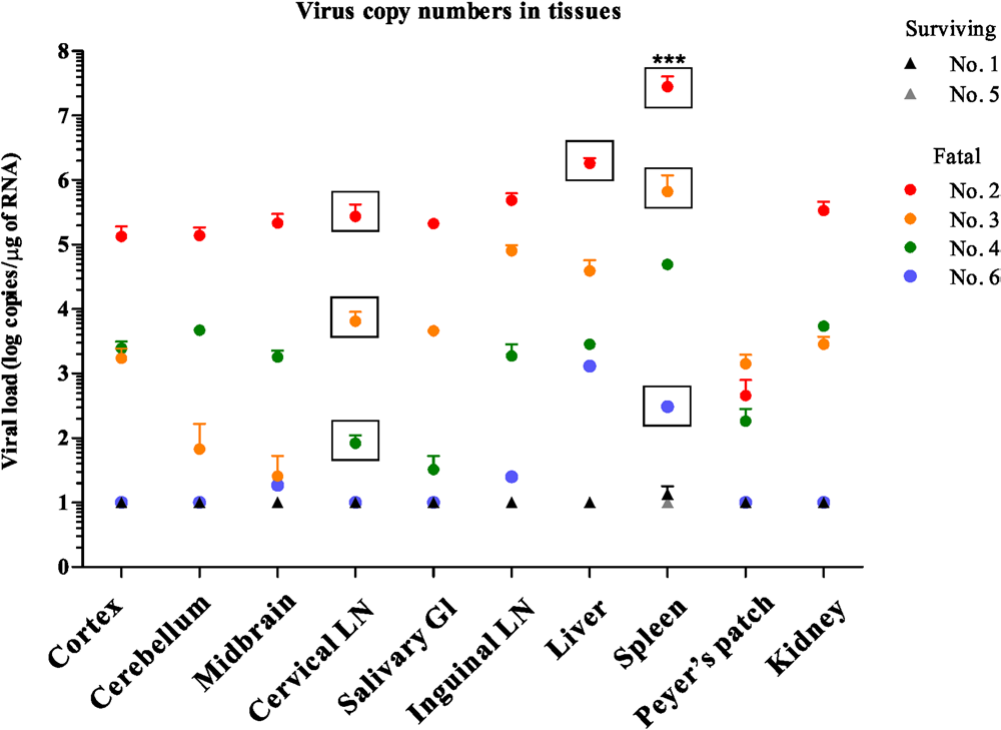
Viral RNA copy numbers of tissues. The SFTSV RNA copy numbers in the tissues of cats were determined by qPCR and calculated as copy numbers per microgram of total RNA. SFTSV was isolated from some organs with high viral load (rectangle). ***P < 0.0001.

SFTSV RNA in the tissues, including the cerebral cortex, cerebellum, midbrain, cervical LNs, salivary gland, liver, spleen, Peyer’s patch and kidney, were detected by qRT-PCR (Fig. 12). A high level of SFTSV RNA was detected in the tissues of fatal cats, especially in the spleen (up to 10^7^ copies/μg of RNA, P < 0.0001). SFTSV RNA was also detected in non-lymphatic tissues, such as the cerebral cortex, cerebellum, midbrain, liver and kidney of the fatal cats. SFTSV was isolated from the tissue lysates with high viral load, such as spleen, salivary gland and cervical lymph node (Figs 12, rectangle and 13). Interestingly, SFTSV was isolated from the cervical lymph node of No. 4 cat with 10^2^ copies/μg of RNA. In these tissues, the round-shaped immunoblast-like cells in the blood vessels were predominantly SFTSV-NP antigen-positive by IHC, so the SFTSV RNA might have been derived from these cells. However, SFTSV RNA was not detected in these tissues of SFTSV-inoculated surviving cats. Thus, we can infer that SFTSV infected the tissues of the SFTSV-inoculated fatal cats, but not in the surviving cats, and proliferated.

**Figure 13:**
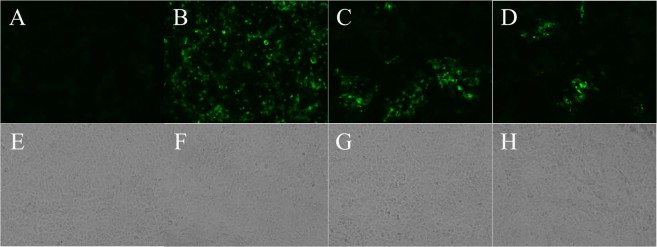
SFTSV isolation from tissues. SFTSV was isolated from spleen, salivary gland and cervical lymph node and confirmed by IF. (**A,E**) Mock (Vero cells). (**B,F**) Spleen (No. 2). (**C**,**G**) Salivary gland (No. 2). **(D**,**H**) Cervical lymph node (No. 4). Magnification of X20. (**E**–**H,A–D**); IF staining; phase contrast image.

**Figure 14:**
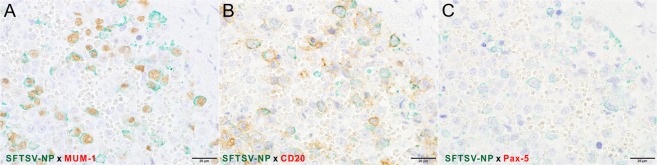
Identification of viral antigen-positive cells in the spleen of a fatal cat No. 2 by double IHC. (**A–C**) Double IHC for SFTSV-NP (green) and the cell markers (brown) with hematoxylin. Viral antigen-positive round-shaped cells are positive for MUM-1 (**A**) and CD20 (**B**). No Pax-5-positive cells exist in this area (**C**). Bars, (**A–C**), 20 µm.

To identify the target cell of SFTSV in the experimentally infected cats, double IHC was performed. The SFTSV-NP antigen-positive round-shaped cells were also positive for MUM-1 and CD20 but not Pax-5 (Fig. 14). In addition, these cells were CD3- and Iba-1-negative (data not shown). The results indicated that SFTSV-NP antigen-positive cells were of B cell lineage at a stage between germinal center B cells and immunoblasts.

**Figure 15:**
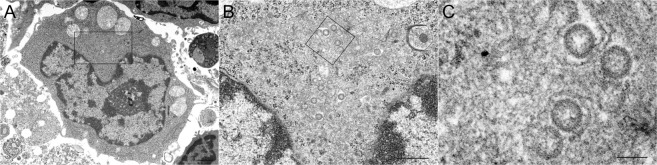
Identification of viral particles in the spleen of fatal cat No. 2 by electron microscopy. (**A–C**) An electron microscopic analysis of the immunoblast-like cells in the cervical lymph node of the SFTSV-inoculated fatal cat (No. 2) at 8 dpi. (**A**) Round-shaped mononuclear cells are seen in the necrotizing lymphatic nodule that appear to be immunoblast cells. Virus-like particles 100 nm in diameter have moderately dense centers and are accumulated in the vacuoles of the immunoblast-like cells (**B**,**C**, higher magnification of **A**). Scale bars indicates 1 µm (**A**), 500 nm (**B**), 100 nm (**C**).

To confirm whether or not SFTSV was actually replicating in the antigen-positive cells, virions were observed by thin-section transmission electron microscopy. Virion particles were found in the vacuoles of the cytoplasm of the immunoblast-like cells in the necrotizing cervical LNs (Fig. 15). This morphology was similar to that observed in DH82 cells^1^. Taken together, these findings strongly indicate that SFTSV preferentially infects immature B cells.

The main lesions in the experimentally SFTSV-infected cats were thus considered to be acute necrotizing lymphadenitis, hemophagocytosis and inflammatory infiltration in the organs lymphatic nodules are located in. These lesions in the fatal cats were quite similar to those in SFTS patients^19^. However, the lesions in the gastrointestinal tract were more severe than those reported in SFTS patients^20^.

## Discussion

In the present study, 0.5- and 2-year-old specific pathogen free (SPF) cats (n = 6) were experimentally infected with SFTSV using the SPL010 strain isolated from an SFTS patient.

Four of the six cats inoculated with SPL010 strain (two 0.5-year-olds and two 2-year-olds) developed severe disease. No relationship was noted between the ages and the disease severity. This conflicts with findings in human SFTS cases, where a majority of fatal SFTS cases occur in patients >50 years of age, making an older age a risk factor associated with fatality of SFTS^17,21^. Recently, the age-associated fatality of SFTSV infection of ferrets were reported^22^. These ferrets were inoculated with similar dose of SFTSV in this study. They discussed that the high fatality rate of aged ferrets might be due to a synergistic effect of high dose of virus and age^22^. Young adult ferrets showed no mortality. On the other hand, young cats (0.5-year-old) showed the most severity of progression and pathogenicity in this study. Thus, these might indicate that cats were more susceptible to SFTS compared to human and ferrets.

In hematology, the WBC, lymphocyte and platelet counts decreased from 1 dpi to 7 or 10 dpi, indicating leukopenia and thrombocytopenia. The cats showed yellowish conjunctiva and dark to black urine. Urobilinogen and bilirubin were positive in urine, and the serum levels of ALT, TBIL and BUN were elevated, indicating hepatic damage or jaundice. However, hepatic damage or degeneration, such as necrosis and inflammation, was not found in the pathological examination. In fatal SFTS cases in humans, the hepatocytes were not infected with SFTSV, suggesting that liver damage in SFTS is induced by a secondary pathological process, such as shock status, hypercytokinemia or hemophagocytosis^19^. In the SFTSV-infected cats, the virus infected cells were predominantly immunoblast-like cells, and parenchymal cells in the liver were not infected, so the liver damage might have been due to a secondary pathological process in our cats, just as in human SFTS cases.

SFTSV RNA were detected in the serum, eye swab, saliva, feces and urine specimens in that order. Furthermore, the viral loads were significantly higher in the SFTSV inoculated fatal cats than in the surviving cats. This indicates that high viral loads were significantly associated with fatal outcome of SFTS in cats, as has been suggested for human SFTS^17,21^. On an autopsy, the LNs were found to be hypertrophied, and extensive hemorrhaging was noted in the GI tract. The pathological examination of the tissues revealed acute necrotizing lymphadenitis and hemophagocytosis, with gastric ulcer, gastritis, enteritis, pancreatitis and pneumonia as secondary lesions. In contrast, the surviving two cats inoculated with SFTSV had no symptoms or signs of SFTS and survived until the end of the experimental period.

In the SFTSV-inoculated fatal cats, the symptoms and signs seemed to resemble those of the fever stage of SFTS. The fatal cats with the most severe symptoms showed a high viral load in the serum as well as GI and hemorrhagic symptoms, although renal failure and MOD were not observed. This suggests that they reached the humane endpoint before the symptoms of the MOD stage appeared.

In the pathological examination, tonsillitis with severe necrotizing lymphadenitis and significant levels of SFTSV-NP-positive cells were observed. In contrast, there were no lesions in the salivary gland, and SFTSV-NP-positive cells were only found in the blood vessels around the salivary gland. This indicates that SFTSV in the saliva or oral cavity originated from the tonsils. Surprisingly, massive amounts of SFTSV RNA were detected in the eye swab sample. The fatal cats had eye discharge, although these ocular tissues were not analyzed in the present study. Such discharge might have been derived from elements of the ocular immune system, such as conjunctiva-associated lymphoid tissue (CALT) or highly vascularized cornea^23^. Indeed, infectious virus was isolated from serum, saliva, eye swab samples and the tissue lysates with high viral load. Blood, saliva and secretory fluid from the eye of the SFTSV-infected cats could be sources of SFTSV transmission to humans. Interestingly, SFTSV was isolated from the tissue with the relatively low viral load. Humans have been considered to be infected with SFTSV by tick bites; however, possible cases of person-to-person transmission of SFTSV have also been reported^13–16^. Thus, there is a risk of direct infection with SFTSV via SFTS-infected animals, such as cats. Actually, several SFTS patients with possible direct transmission from SFTS-contracted cats were reported in Japan.

In the SFTSV-inoculated cats, SFTSV-NP-positive cells were immunoblast-like cells that were MUM-1 and CD20-positive but Pax-5 negative, indicating that the major target cells in the cats were post-germinal center B cells^24–29^. Why these immunoblast-like cells propagate upon infection with SFTSV in cats is unclear at present. In fatal SFTS patients, SFTSV-NP antigen-positive cells have been shown to be atypical lymphoid cells not only in the LNs and spleen but also in many organs or tissues^19^. It will therefore be interesting to determine whether or not the major target cells in humans are also in the B cell lineage.

In this study, functional neutralizing antibodies were not detected in the fatal cats, consistent with the deficiency of the clearance of SFTSV, although IgM and IgG against SFTSV-NP were detected by ELISA and IF. This phenomenon has been reported in Ebola virus-infected patients and SFTSV-infected patients^30,31^. This defective functional humoral response could be caused by the damage of B cells, such as plasmablast due to the target of SFTSV and the breakdown of the immune balance by extensive apoptosis or necrosis of lymphocytes and monocytes. Recent report has been suggested that proliferated plasmablast could not secret functional neutralizing antibodies because of the inhibition of class-switch recombination (CSR) of mature B cells^30^. This could be plausible since immunoblast-like cells, such as plasmablast, proliferated characteristically in our study, which further studiese are necessary. It was indicated that the failure to induction of neutralizing antibodies affect the fatality of SFTSV infection in cats.

Hemophagocytosis was prominent in the spleen, LNs and bone marrow and appeared in many other organs as well. The hemophagocytic macrophages were negative for SFTSV-NP. In some SFTS patients, hemophagocytic lymphohistiocytosis (HLH) has been reported, and hemophagocytic macrophages have been shown to be SFTSV-NP-positive^32^. In a mouse model of SFTS, it was reported that SFTSV adhered to platelets and facilitated phagocytosis by macrophages, proliferating within macrophages^3^. In the present study, the hemophagocytic macrophages were SFTSV-NP-negative. It is uncertain whether the SFTSV adhered to platelets and SFTSV-adhered platelets were engulfed by macrophages.

It has been suggested that cytokine storm can cause hemophagocytosis^33^. The level of pro-inflammatory cytokines and chemokines was elevated in the fatal cats at 7 dpi, then reduced at 8 dpi. Lots of inflammatory cells and lymphatic tissues were damaged at 8 dpi in pathological examination. Thus, these results could reflect the moribund status of these cats at 8 dpi. Interestingly, the levels of cytokines relating to B cell proliferation and immunoglobulin production were elevated in one fatal cat (No. 6). This cat had a high titer of neutralizing antibodies at 10 dpi (1:40). In addition, the level of IL-2, relating to regulatory T cell, reduced at 3 dpi, then followed by the elevation to 10 dpi. The copy numbers of viral RNA in tissues were the lowest among fatal cats. And some clinical signs were the mildest among fatal cats. These result might indicate that the antibody-associated cytokine could affect the severity of pathogenicity of SFTSV in cats, although further studies are necessary. Other cytokines and chemokines in fatal cats were not significantly different from those of surviving cats. For the cytokine and chemokine examination, samples were inactivated by UV and treated with SDS and Triton X100 because SFTSV is the pathogen that should be treated in the biosafety level 3 facility. This could affect the results of cytokine and chemokine levels. Since the level of cytokines in naturally occurred SFTS cat cases was extensively high by same protocol (data not shown), results are thought to be credible. Also, the variances of cytokine and chemokine levels could be caused by the individual differences.

In a lethal SFTS animal model using interferon-α/β receptor knock-out (IFNAR^−/−^) mice, IbaI-positive macrophages and Pax5-positive immature B cells in the spleen and LNs were shown to be infected with SFTSV^34^. Hemophagocytosis was not observed in the spleen, LNs, bone marrow or other tissues in the lethal mouse model, although hemophagocytosis is a characteristic lesion in fatal cases of SFTS in humans and cats experimentally infected with SFTSV. Thus, the pathogenesis of SFTSV infection in IFNAR^−/−^ mice differs slightly from that in human and cats. In contrast, a lethal SFTS animal model using ferrets was similar to human and cat SFTS^22^. It is interesting to clarify SFTS susceptible animals upon SFTSV infection in future.

Hemophagocytic syndrome has been reported in dogs and cats as a primary or secondary disease, including infection, neoplasia and idiopathic diseases^35,36^. Given that hemophagocytic macrophages and affected lymphocytes are not atypical, the hemophagocytosis in the SFTSV-inoculated cats is thought to be virus-associated hemophagocytic syndrome, similar to cases of parvovirus B19, Epstein-Barr virus and cytomegalovirus^37–39^.

In conclusion, we herein demonstrated that SFTSV caused a lethal infection in cats, confirming that these animals are susceptible to SFTSV infection. Further studies are necessary to elucidate the pathogenesis of SFTS in cats.

## Material and Methods

### Viruses and animals

SPL010 strain of SFTSVwas used in the present study (Table 3). SPL010 (Genbank accession No. AB817983, AB817999 and AB817991) was isolated from a SFTS patient in 2013 in Japan. The viruses were propagated in Vero cells (ATCC # CCL-81).

**Table 3:** Information of cats.

No. of cat	Breed	Age (year)	Sex	Inoculated virus	Dose of virus (ffu/2 mL)	Route of inoculation
1	Russian Blue	0.5	♀	SPL010	1 × 10^7^	i.v.
2	American Shorthair	0.5	♀	SPL010	1 × 10^7^	i.v.
3	American Shorthair	0.5	♀	SPL010	1 × 10^7^	i.v.
4	American Shorthair	2	♀	SPL010	1 × 10^7^	i.v.
5	American Shorthair	2	♀	SPL010	1 × 10^7^	i.v.
6	Russian Blue	2	♀	SPL010	1 × 10^7^	i.v.

SPF cats that were healthy and serologically negative for SFTSV were obtained from HAMRI Co., Ltd. (Tokyo, Japan; 0.5-year-old cats) and NAS Laboratory Co., Ltd. (Narita, Japan; 2-year-old cats). The animals were housed in separate cages at the Biosafety Level 3 laboratory of the National Institute of Infectious Diseases (NIID, Musashimurayama, Japan).

### Ethical statement

The experiments with animals were performed in strict accordance with the Animal Experimentation Guidelines of the NIID. The protocol was approved under Permission Number 117103 by the Institutional Animal Care and Use Committee of the NIID.

### Animal infection and sample collection

Two groups each of 0.5-year-old and 2-year-old cats (n = 3 per group) were inoculated intravenously (i.v.) with 10^7^ TCID_50_/mL of SFTSV SPL010 strain (1 group) (Table 3) and then monitored for 2 weeks after inoculation. The humane endpoints were set as following points: loss of appetite (more than 4 days), weight loss of 20%, fever of 40 °C or higher followed by body temperature reduction, reduction of WBC (below 5,000/μL) and platelet (below 100,000/μL) and jaundice of visible mucosa or serum. When cats meet more than two points, they were considered to have reached the humane endpoint, then anesthetized and had their blood removed by cardiac puncture for sacrifice^40^. These cats were considered ‘fatal’. At 0, 1, 3, 7, 8, 10 and 14 days post-inoculation (dpi), clinical examinations including measurements of the body weight and temperature were performed. Blood was drawn, and saliva, eye swab, rectal swab and urine samples were obtained from the anesthetized cats. Eye and rectal swabs were suspended in 0.5 mL DMEM supplemented with 5% FCS and kanamycin and centrifuged at 10,000 rpm for 5 min; supernatants were stored at −80 °C. Serum was separated by centrifugation and stored at −80 °C. Saliva was obtained by Salivette^Ⓡ^ (Sarstedt, Nümbrecht, Germany) and centrifuged at 3,000 rpm for 5 min. Collected saliva samples were then stored at −80 °C. Urine samples were stored at −80 °C.

### Blood cell count

Collected blood was subjected to a complete blood cell (CBC) count analysis. Counts of red blood cells (RBCs), white blood cells (WBCs) and platelets, and hemoglobin, hematocrit, mean corpuscular volume (MCV), mean corpuscular hemoglobin (MCH) and MCH concentration (MCHC) were measured using an automated blood cell counter. An examination of stained blood smears was also performed to evaluate the differential count of neutrophils, lymphocytes, monocytes, eosinophils and basophils by a microscopic analysis.

### Measurement of cytokine and chemokine by luminex assay

Cytokines and chemokines in cat sera were measured using a Luminex Milliplex^Ⓡ^ MAP multiplex assay (FCYMAG20KPX19BK; Millipore, Darmstadt, Germany). SFTSV in the sera was inactivated by ultraviolet irradiation for 10 min. Samples were then mixed with the same volume of cell extraction buffer with SDS (final concentration 0.05%) and Triton X100 (final concentration 0.5%) supplied in the kit and incubated for 10 min at 4 °C. The following cytokines and chemokines were measured using the assay: first apoptosis signal (Fas), Fms-related tyrosine kinase 3 ligand (Flt-3L), granulocyte-macrophage colony-stimulating factor (GM-CSF), IFN-γ, IL-1β, IL-2, platelet derived growth factor (PDGF-BB), keratinocyte chemoattractant (KC), stromal cell-derived factor 1 (SDF-1), regulated upon activation normal T cell expressed and secreted (RANTES), stem cell factor (SCF), monocyte chemotactic protein-1 (MCP-1), TNF-α, IL-18 IL-12p40, IL-13, IL-4, IL-6 and IL-8.

### Biochemical examinations

Sera were used to determine the blood biochemical values. A Vetscan II automated analyzer (Abaxis, Tokyo, Japan) equipped with a multirotor II VCDP (Abaxis) was used to measure the albumin (ALB), alkaline phosphatase (ALP), alanine aminotransferase (ALT), (AMY), total bilirubin (TBIL), blood urea nitrogen (BUN), total calcium (CA), phosphorus (PHOS), creatinine (CRE), glucose (GLU), NA^+^, K^+^, total protein (TP) and globulin (GLOB).

### Urine examinations

Urine samples were analyzed semi-quantitatively with a dipstick test (Uropaper III EIKEN, Eiken Chemical Co., Ltd., Tokyo, Japan) according to the manufacturer’s instructions. Urobilinogen, occult blood, bilirubin, ketone body, glucose, protein, pH and specific gravity were measured.

### Isolation and detection of SFTSV RNA by quantitative one-step reverse transcription polymerase chain reaction (qRT-PCR)

A total of 100 μL of serum, saliva, eye swab, rectal swab and urine specimens was subjected to RNA extraction using a High Pure Viral RNA Kit (Roche, Mannheim, Germany). RNA was extracted from 20–30 mg of tissue using ISOGEN (Nippon Gene, Tokyo, Japan). RNA extraction was performed according to the manufacturer’s instructions. qRT-PCR with extracted RNA was performed according a previous report^41^.

### Virus isolation

Serum, saliva, urine, eye swab and rectal swab specimens were subjected to the virus isolation, using Vero cells. Briefly, 50 μL of samples were inoculated into Vero cells, then, DMEM with 2% fetal bovine serum were added, followed by cell culture at 37 °C in 5% CO_2_ for up to one week. Ten % tissues homogenates were prepared in DMEM with 2% fetal bovine serum. Then, they were inoculated onto Vero cells and cultured at 37 °C in 5% CO_2_ for up to one week. When cytopathic effects (CPE) appeared, cells were harvested and centrifuged at 14,000 rpm for 2 minutes and the supernatants were stored in −80 °C. In case that CPE did not appear, the cells were passaged until 5 times. To confirm the virus, CPE-appeared cells were observed by immunofluorescence staining using rabbit antibodies against SFTSV-NP.

### Detection of IgM and IgG in cats by an enzyme-linked immunosorbent assay (ELISA)

IgM and IgG antibodies against SFTSV in the cat sera were determined by an ELISA. Serum samples were inactivated at 56 °C for 1 h. the ELISA was performed as described previously with slight modifications^42^.

In brief, each well of the ELISA plate (Nunc-Immuno^TM^ plate; Thermo Fisher Scientific, Roskilde, Denmark) were coated with SFTSV- or mock-infected Huh7 cell lysates at 4 °C overnight and then blocked with 20% Blocking One in PBS (blocking solution) (Nacalai Tesque, Inc., Kyoto, Japan) at room temperature for 1 h. Cat sera were serially diluted 4-fold from 1:100 to 1:6400 in blocking solution and incubated with antigens at 37 °C for 1 h. Secondary antibodies against feline IgM (horseradish peroxidase [HRP]-conjugated goat anti-feline IgM; Novus Biologicals, Littleton, Colorado, USA) and feline IgG (HRP-conjugated goat anti-feline IgG; Novus Biologicals) diluted to 1:2,000 in blocking solution were incubated at room temperature for 40 min. The reaction was then visualized by a substrate for HRP, ABTS (2,2azinobis (3-ethylbenzthiazolinesulfonic acid); Roche) for 30 min at room temperature, after which the optical density (OD) at 405 nm was measured with a reference at 490 nm by a microplate reader (BIO-RAD, Tokyo, Japan). The OD of mock-antigen was subtracted from the OD of SFTS-antigen.

The cut-off value was set as the average subtracted value plus three times the standard deviation (SD; mean + 3 SD). The cat sera were considered positive if the OD at 405 nm was above the cut-off value.

### Detection of IgG in cat serum by Immunofluorescence assay (IF)

For the detection of IgG by IF, IF slides containing SFTSV-infected cells were prepared. Briefly, SFTSV were inoculated into Vero cells (ATCC) and incubated at 37 °C in 5% CO_2_. At 3dpi, they were harvested and mixed with Vero cells at 1:1, spotted on 14-well HT-coated slide glasses (AR Brown, Tokyo, Japan), air dried with UV irradiation and fixed with acetone at room temperature for 5 min and stored at −80 °C. Then, IF was performed to detect IgG against SFTSV in cat sera, according to the previous study^43^. The Ab titers of tested cat sera were recorded as the reciprocals of the highest dilutions producing positive staining.

### Fifty percent-plaque reduction neutralization titer (PRNT50)

PRNT assay was used to determine the neutralizing antibodies against SFTSV with Vero cells (ATCC). Approximately 40 plaque-forming units of YG1 (ref) strain of SFTSV were mixed with serially-diluted heat-inactivated sera and incubated for 1 h at 37 °C, then inoculated into confluent monolayers of Vero cell in 12-well plates for 1 h at 37 °C. Then the inoculums were removed and the cells were washed once with DMEM containing 2% FBS and kanamycin and cultured at 37 °C in 5% CO_2_ in DMEM containing 2% FBS, kanamycin and 1% methylcellulose for one week. Plaques were detected as described previously^44^. In brief, cultured cells were fixed with 10% buffered formalin and exposed to UV light to inactivate virus. The cells were treated with 0.1% Triton X-100, followed by staining with rabbit antibodies against SFTSV-NP as primary antibodies and HRP-conjugated recombinant protein A/G (Cat. No. 32490, Thermo Scientific, Rockfore, USA) as secondary antibodies. The plaques were visualized with 3, 3′-diaminobenzidine tetrahydrochloride (Peroxidase stain DAB kit (Brown stain), Nacalai Tesque, Kyoto, Japan). The values of PRNT50 were determined as reciprocal of the highest dilution at which the number of the plaques was <50% of the number calculated without cat serum.

### Pathological examinations

The macroscopic findings were first examined for the euthanized cats, after which the brain, spinal cord, lymph nodes, salivary glands, tonsil, sternal bone marrow, gastrointestinal tract, heart, thymus, lungs, spleens, livers, adrenal gland and kidneys were collected for histopathological examinations. The tissues were fixed in 10% buffered formalin and then routinely processed and embedded in paraffin, sectioned and stained with hematoxylin and eosin (H&E).

Immunohistochemical staining (IHC) was also performed to detect SFTSV nucleoprotein (NP) antigens in the paraffin-embedded sections, as previously described^4,45^. For IHC, antigens were retrieved by hydrolytic autoclaving in citrate buffer (pH 6.0) for 10 min at 121 °C. After endogenous peroxidase quenching with a 0.3% solution of H_2_O_2_ in methanol, IHC was then performed using a rabbit polyclonal antibody against SFTSV NP^45^ as a primary antibody, and the polymer-based detection system Nichirei-Histofine Simple Stain MAX-PO^®^ (Nichirei Biosciences, Inc., Tokyo, Japan) was used to visualize the antigens.

After reaction of chromogen with diaminobenzidine (DAB), the nuclei were counterstained by hematoxylin. To identify the cells infected with SFTSV, double IHC was performed using anti-MUM-1 rabbit monoclonal antibody (specific to germinal center B cells located in the light zone and plasma cells; cat No. ACR352A; Biocare Medical, CA, USA), anti-CD20 rabbit antibody (specific to B cells; cat No. RB-9013-P0, Thermo Scientific, Waltham, MA, USA) and anti-Pax-5 monoclonal antibody (specific to pro-, pre- and mature B cells but not to plasma cells; cat No. 610863; BD Biosciences, Sa Diego, CA, USA) as primary antibodies^24–29^. A DAB Chromogen Kit (Biocare Medical) and Vina Green Chromogen Kit (Biocare Medical) were used as chromogens for the HRP visualization. Following the first staining for each cell marker using the polymer-based detection system with DAB, denaturing was performed by hydrolytic autoclaving in citrate buffer (pH 6.0) for 10 min at 121 °C. The second staining was performed for viral antigens with Vina Green. Nuclei were counterstained by hematoxylin for 10 sec.

### Electron microscopy

A cervical LN was obtained from a SPL010-inoculated non-surviving cat. The tissue was fixed with buffered 2.5% glutaraldehyde-2% paraformaldehyde in 0.1 M phosphate buffer (pH 7.4) for 2 h at room temperature, post-fixed in 2% osmium tetroxide, and embedded in Epon. Ultrathin sections were stained with uranyl acetate and lead citrate and observed under a transmission electron microscope (HT7700; Hitachi High Technologies, Tokyo, Japan) at 80 kV.

### Statistical analysis

Data were analyzed using unpaired t test with Welch’s correction. Graph Pad Prism 5 for Windows (GraphPad Software, San Diego, California, USA) was used to perform the statistical analysis. A 95% confidence interval and a 5% level of significance were adopted. P values ≤ 0.0001 (***) and 0.005 (**) were considered to be significant.

## Data Availability

The datasets generated during the current study are available from the corresponding author on reasonable request.
